# Non‐Surgical Treatment of Tracheal Glomus Tumour Using Rigid Fiberoptic Bronchoscopy: A Case Report

**DOI:** 10.1002/rcr2.70236

**Published:** 2025-06-19

**Authors:** Haanieh Nasiraei Mir, Maryam Mazraehei Farahani, Arda Kiani, Somayyeh Ghadimi

**Affiliations:** ^1^ School of medicine Tehran University of Medical Sciences Tehran Iran; ^2^ Farhikhtegan Medical Convergence Sciences Research Center, Farhikhtegan Hospital Tehran Medical Sciences, Islamic Azad University Tehran Islamic Republic of Iran; ^3^ Clinical Tuberculosis and Epidemiology Research Centre, National Research Institute of Tuberculosis and Lung Diseases (NRITLD), Shahid Beheshti University of Medical Sciences Tehran Iran; ^4^ Lung Research and Developmental Center, Shahid Beheshti University of Medical Sciences Tehran Iran

**Keywords:** carina, fiberoptic bronchoscopy, non‐surgical treatment, tracheal glomus tumour

## Abstract

Glomus tumours are a group of benign neoplasms originating from the modified smooth muscle cells at the arteriovenous anastomosis that can impact body temperature and blood flow. They are most commonly seen in the subungual region of the subcutaneous tissue, and less frequently seen in organs including the lung and trachea. Tracheal Glomus Tumours (TGTs) are extremely rare subtypes of primary tracheal tumours affecting patients with a mean age of 45, most commonly seen in males and presenting symptoms including cough, dyspnoea, or haemoptysis. Although surgery is considered the primary approach for the treatment of TGT, alternative approaches such as rigid fiberoptic bronchoscopy are considered especially due to the rarity of this disease. In the present case, we report a 38‐year‐old male patient with a TGT near the carina with symptoms including dyspnoea, cough, and mild haemoptysis. Although the diagnostic approaches revealed a polyploid mass, surgery was related to a higher risk of complications and thus, we used rigid fiberoptic bronchoscopy for the resection of the tumour and considered a 12‐month follow‐up for monitoring of the patient to assess the need for potential surgical treatment.

## Graphical abstract

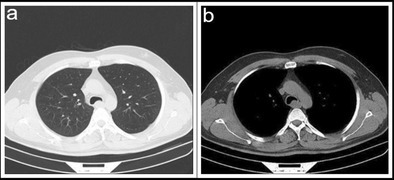

## 1 Introduction

Glomus tumours (GTs) are distinctive and generally benign neoplasms that originate from the formation of modified cells of smooth muscle within typical glomus bodies at the arteriovenous anastomosis and can affect body temperature and blood flow [1, 2, 3]. Tracheal Glomus Tumour (TGT) is an unusual and extremely rare subtype of primary tracheal tumours, which account for less than 0.1% of all malignancies [1, 4]. The mean age of these tumours is 45 years old, and they are more commonly seen in males, with a ratio of 2:1 compared to 7:1 in females [5]. Symptoms of TGT vary from not presenting any to having severe discomfort, but patients typically present nonspecific symptoms, including cough, dyspnoea, or haemoptysis [3, 6]. However, most cases of TGTs become symptomatic, and therefore, an intervention is necessary [7]. The choice of treatment is considered surgery, including thoracotomy and bronchoscopic electrocautery, but there are ideal evaluations of the treatment reported by few studies [6]. Rigid bronchoscopy is a diagnostic and therapeutic method for the treatment of most endobronchial pathologies such as endobronchial tumours [8]. Due to the rarity of TGTs, there is not much data available regarding these tumours, and not many cases reported [9]. In the present case, we report a patient with TGT in the carina and the rigid fibreoptic bronchoscopy as an alternative treatment rather than surgery according to the potential risks of surgical operation.

## 2 Case Report

The 38‐year‐old male patient with no past medical history was referred to Masih Daneshvari Hospital in Tehran, Iran.

He had a two‐month history of persistent cough, occasional non‐massive haemoptysis, and Class II functional dyspnoea. Upon thorough evaluation, his physical examination and vital signs were largely unremarkable. The results of the patient's laboratory tests were within the normal range. His echocardiography showed an ejection fraction of 55% and 20 mmHg of pulmonary artery pressure.

**FIGURE 1:**
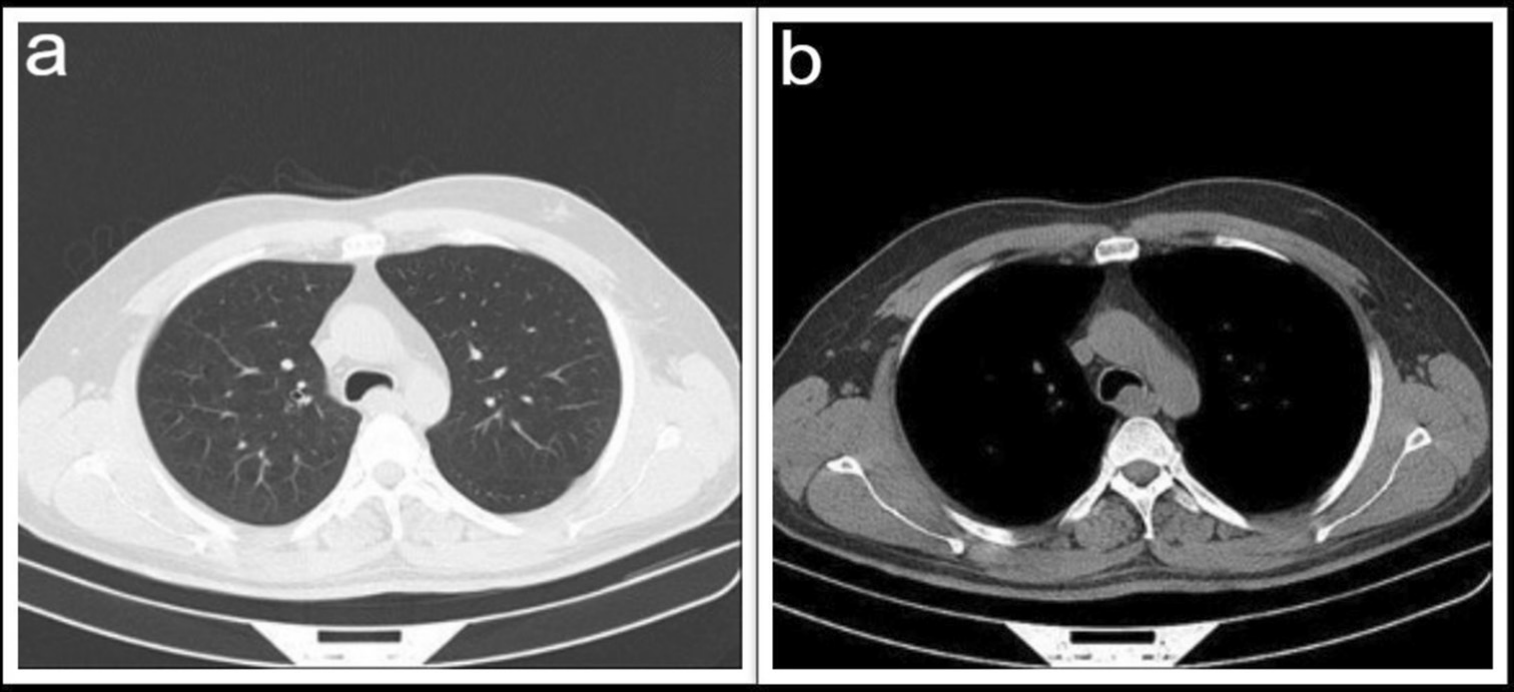
(a) Tracheal tumour parenchymal view. (b) Tracheal tumour mediastinal view.

A computed tomography (CT) scan of the chest was performed for him and showed a huge mass in the distal trachea, approximately 1 cm above the carina (Figure 1).

**FIGURE 2:**
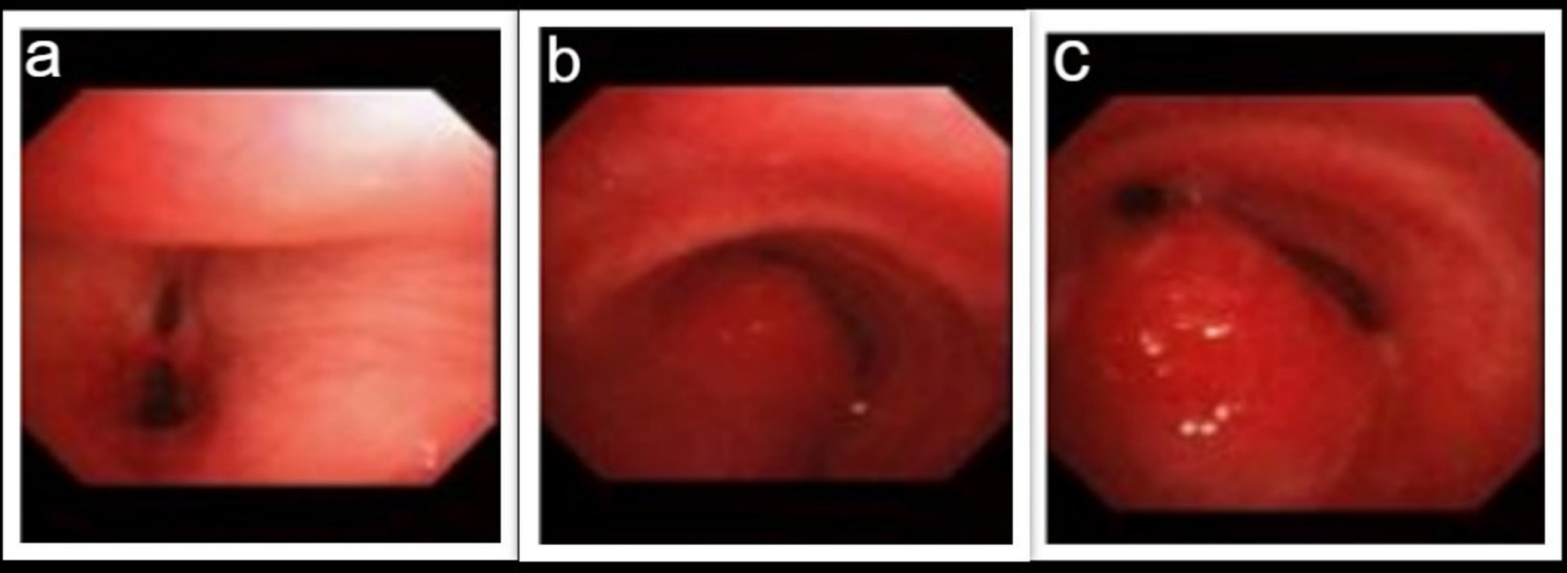
Bronchoscopy. (a) vocal chords, (b) mid to distal portion of trachea, (c) distal portion of the trachea, an obstruction by a mass is seen.

Given the findings, the patient became a candidate for bronchoscopy. The procedure was conducted under general anaesthesia. The examination unveiled a large polypoid mass in the distal trachea, precisely 1 cm above the carina (Figure 2). A biopsy was performed, and the specimen was sent to the pathology department for further analysis.

After 5 days, the pathology results were received. The irregular, tan‐brown soft tissue biopsy, measuring 1.5 cm × 1.3 cm × 0.7 cm, confirmed the presence of a hypervascular low‐grade benign neoplasm consistent with a glomus tumour (glomangioma) (Table 1).

**TABLE 1:** Results of immunohistochemical markers in tumour biopsy specimens.

IHC marker	Result
Chromogranin	Negative
Synaptophysin	Negative
AE1/AE3	Negative
TTF1	Negative
P63	Negative
CD117	Negative
MSA	Diffusely positive
Desmin	Negative
Ki67	Less than 5%

Given the anatomical location of the tumour, particularly its proximity to the carina, surgical intervention included significant risks. Consequently, a conservative approach was favoured, opting for a non‐surgical intervention to mitigate potential complications.

**FIGURE 3:**
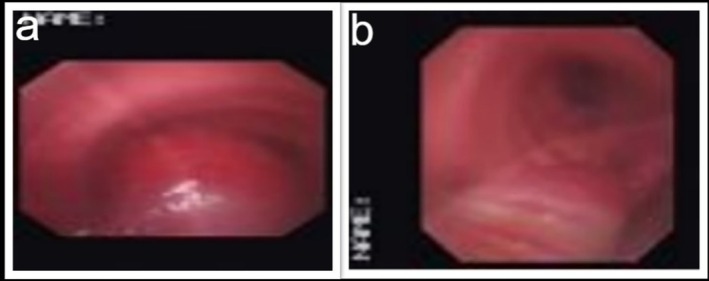
Bronchoscopy for tumour resection: (a) tumour at the distal portion of the trachea, (b) distal portion of trachea after complete resection.

The patient was referred to the bronchoscopy department, where the procedure was conducted under general anaesthesia, and a 12 mm Storz medical rigid bronchoscope was carefully inserted. The tumour was successfully debulked in its entirety, and any bleeding was successfully controlled (Figure 3). Following the intervention, the patient was moved to the recovery area to be monitored.

A follow‐up plan was established in light of the tumour's characteristics and the patient's condition. A comprehensive 12‐month follow‐up was scheduled to assess surgical options should any significant symptoms, such as massive bleeding, worsening dyspnoea, or new mass formations, arise. This proactive approach ensures that potential complications are addressed while promptly providing ongoing patient health support.

**FIGURE 4:**
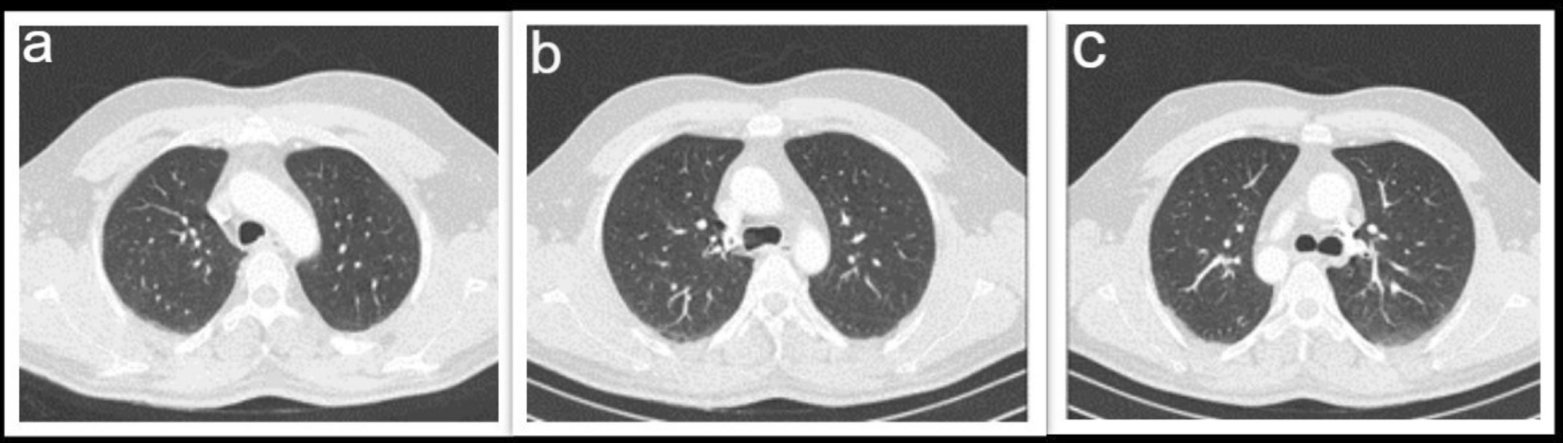
CT scan after 12 month follow up (a) mid portion of trachea, (b) distal part of trachea above the carina, (c) trachea in the level of carina.

After 12 month follow up, the patient was stable with no complaint of haemoptysis, dyspnoea, or hoarseness. His CT scan showed no tumoural lesion in the trachea (Figure 4).

## 3 Discussion

In this case, we performed a non‐surgical technique using rigid fiberoptic bronchoscopy to resect a glomus tumour located near the Carina, which could raise operational risks in surgery for the patient. Primary tracheal tumours are among the rarest tumours, with the majority being squamous cell carcinoma or adenoid cystic carcinoma and the minority being mesenchymal‐originated tumours [10]. Glomus tumours are sporadic and typically benign mesenchymal neoplasms and distinctive types of vascular tumours that are characterised by modified smooth muscle cells resembling the glomus body and comprise less than 2% of all soft tissue tumours [10, 11, 12]. At the ultrastructural level, tumour cells are observed to resemble epithelioid smooth muscle cells, distinguished by a surrounding basement membrane, the presence of pinocytotic vesicles associated with the plasma membrane, and the existence of cytoplasmic myofibrils [10]. They also contribute to regulating the blood flow and temperature of the body due to their origin from the glomus cells surrounding the arterial and venous anastomosis in the subcutaneous tissues and dermal area. It is claimed that there is uncertainty about the aetiology of these tumours, and it can be associated with three main factors, including trauma, endocrine disease, or the inheritance of an autosomal dominant trait. GTs may occur due to somatic or germline mutations with the association of the gene with chromosome 1p21–22.3 in the inherited variant [11]. Based on differences at this level and the proportions of vascular structures, glomus cells, and smooth muscle tissue in the tumour, it has been classified into four distinctive subtypes: classic glomus tumours in 75% of cases, glomangiomas in 20% of cases, and glomangiomyomas and oncocytic glomus tumours each in few cases [13].

Glomus tumours can occur in all portions of the body, most commonly seen in the deep dermis and the near subcutaneous tissue, especially the subungual area, and uncommonly seen in the stomach, mediastinum, penis, vagina, lung, and trachea where the glomus bodies are not present [10, 11, 13].

Tracheal Glomus Tumours (TGTs) are tightly lined up round or oval‐shaped tumours, located in the tracheal mucous membrane, arising most commonly from the lower two‐thirds of the trachea in the lower wall where there are numerous vessels and mucus glands [5, 14, 15]. The cells demonstrate strong positive staining for vimentin (VIM), smooth muscle actin (SMA), and type IV collagen while showing negative results for epithelial and neuroendocrine markers such as cytokeratin (CK), chromogranin, S‐100 protein, and desmin [15]. TGT can be asymptomatic, but in symptomatic patients, symptoms include dyspnoea in most cases, cough and wheezing, haemoptysis, chest pain, and fever [14, 15].

The primary treatment modality for this tumour is surgery, accounting for 67.1% of cases, and other choices mentioned in the literature include endoscopic tumour removal, segmental resection of the trachea accompanied by an end‐to‐end anastomosis, tracheal sleeve resection, wedge resection of the trachea, longitudinal resection with spiral tracheoplasty, endobronchial interventions, and bronchoscopic GT resection [9, 15].

In most cases, surgical resection is applied, and regarding the evidence of the three‐month to six‐year follow‐up, there was no report of recurrence. According to a study by L. Sakr et al., in three patients, complete endoscopic tumour removal with the help of laser for mechanical removal by rigid bronchoscopy has been reported. Successful outcomes have been presented, and there was no report of tumour recurrence in 2 of them according to 12 and 24‐month follow‐ups [5].

Indication of the rigid bronchoscopy includes diagnostic indications such as obtaining the biopsy of large tissues and therapeutic indications such as complex foreign body removal, massive haemoptysis management, and obstruction treatment of central airways because of malignant or non‐malignant causes. This method is considered an easy and safe procedure which is commonly used for the treatment of most endobronchial pathologies such as obstruction of the airway due to aspiration of the foreign body, bronchial or tracheal stenosis, endobronchial tumours, and massive haemoptysis [8, 16]. Rigid bronchoscopy is commonly also used along with other endobronchial tools such as tracheobronchial stents, photodynamic therapy, and laser [8]. The ideal method is doing the rigid bronchoscopy with a fibreoptic bronchoscope which is passed through the rigid tube [17].

As claimed in the literature, according to the higher potential of recurrence using endoscopy rather than surgical resection of the tumour, the preferred method for the treatment is surgery [9]. However, in the present case, according to the tumour's location, the decision to operate a non‐surgical intervention was made.

The tumour was near the Carina, which is considered challenging for the resection of tracheal tumours [18]. Therefore, we operated rigid fibreoptic bronchoscopy for the resection of the tumour, which was easier for the surgeon and could lower the potential surgical risk for the patient. The 12‐month follow‐up showed post‐operative complications and no tumour recurrence.

## Author Contributions

**Haanieh Nasiraei Mir:** prepared the initial draft of the manuscript. **Maryam Mazraehei Farahani:** contributed to manuscript revision and patient data management. **Arda Kiani:** revised the manuscript and provided patient data. **Somayyeh Ghadimi:** revised the manuscript and contributed to patient data management.

## Ethics Statement

The authors declare that appropriate written informed consent was obtained for the publication of this manuscript and accompanying images.

## Conflicts of Interest

The authors declare no conflicts of interest.

## Data Availability

Data sharing is not applicable to this article as no new data were created or analysed in this study.
